# A hybrid MCDM approach based on combined weighting method, cloud model and COPRAS for assessing road construction workers’ safety climate

**DOI:** 10.3389/fpubh.2024.1452964

**Published:** 2024-09-26

**Authors:** Jing Cai, Yihui Hu, Yiming Peng, Fengxiang Guo, Jian Xiong, Ran Zhang

**Affiliations:** Faculty of Transportation Engineering, Kunming University of Science and Technology, Kunming, China

**Keywords:** safety climate, combined weighting method, cloud model, COPRAS, construction worker safety

## Abstract

**Purpose:**

The purpose of this paper is to propose a novel approach to assess the safety climate level of different groups of workers in a construction company and predict safety performance and implement targeted improvement measures.

**Design/methodology/approach:**

This paper utilizes the BP neural network and random forest algorithm to establish a weight learning mechanism for calculating the weights of safety climate evaluation criteria. The cloud model is employed to construct the decision matrix for different groups under the evaluation criteria. Meanwhile, the paper utilizes the COPRAS method to compare the safety climate of different groups.

**Findings:**

The findings show the accuracy of the CM-COPRAS model is assessed by comparing it with the other methods. The three models are almost consistent in assessing the safety climate for working age groups, accident experience groups, and work type groups, with slight differences in the evaluation results for the education groups. The consistency of the computational results of the CM-COPRAS model with the results of the existing research, i.e., that the education level is positively proportional to the safety climate supports the reasonableness and validity of the CM-COPRAS model.

**Originality:**

The paper proposes a hybrid MCDM method that integrates the Combined weighting method, Cloud model, and COPRAS for safety climate level evaluation in different construction worker groups. A case study is presented to demonstrate the applicability of the proposed method and to compare it with other methods to validate the effectiveness of the present method.

## 1 Introduction

Safety climate is generally defined as the shared perception of workgroup members regarding safety-related policies, procedures, and practices (1). The concept of safety climate is introduced in the realm of construction safety management, with the aim of predicting safety performance (2). The safety climate has a positive impact on the overall safety management of Chinese construction companies (41). Recently, more and more construction safety managers have conducted research on evaluating safety climate level as a predictor of construction safety performance [e.g., (3–5)]. As a shared perception of safety within a group, safety climate can be influenced by individual personality traits and the workplace. For instance, researchers have identified various demographic variables that can affect safety climate, such as age (6), gender (7), education (8), occupation (9), religion (10), and nationality (11). These demographic factors can lead to biases in awareness, behavior, and attitudes of different groups towards specific safety issues. Consequently, when assessing safety climate, it is essential to consider demographic attribute variables of various groups. Compared with the overall organizational safety climate, the group safety climate has a stronger and more direct predictive effect on the safety performance of the construction industry organization (12). When assessing the safety climate in the construction industry, comparing the safety climate scores of different demographic groups, such as organizations, age, workgroup types, and worker experience, is of crucial importance (13).

While the significance of considering the intensity and level of safety climate among different groups in the construction industry has been acknowledged, most of the current studies on evaluating safety climate in this field do not take into account the differences among groups [e.g., (13–16)]. The evaluation methods primarily relied on factor analysis of questionnaire scales, making it challenging to explore the characteristic safety climate of diverse groups. Furthermore, these studies lacked effective theoretical support and strategic approaches for enhancing actual safety measures, particularly regarding group behavior improvement. Undertaking a systematic study to identify a suitable method for assessing the level of safety climate among various groups of workers and elucidating the differences in safety climate among these groups is imperative. This endeavor may enable the design of comprehensive safety interventions tailored to specific groups, consequently identifying opportunities for improving workplace safety and health and facilitating managers in implementing targeted improvement strategies.

To address the practical needs mentioned above, this paper proposed a hybrid multi-criteria decision-making (MCDM) approach that combines the combined weighting method, Cloud model, and COPRAS for assessing the safety climate level of road construction workers among different groups. The combined weighting method, utilizing the Maximizing Deviations approach, was employed to determine the weights of safety climate evaluation indicators. This was achieved by integrating the results obtained from the BP neural network and Random Forest. The cloud model method was utilized to generate decision matrices that captured the numerical characteristics of sample data from different construction worker groups within a stochastic and fuzzy environment. The COPRAS method was applied to rank and compare the safety climate levels among the different construction worker groups. This approach helps in identifying the groups that perform better or worse in terms of safety climate, facilitating targeted interventions and improvements.

The structure of this paper is as follows: Section 2 presents a comprehensive review of the literature related to safety climate evaluation criteria and MCDM methods. Section 3 provides a detailed explanation of the hybrid MCDM method proposed in this study. In Section 4, a real-world case study is presented to showcase the practicality of the proposed framework and demonstrate the effectiveness of the implemented procedures and algorithms. Finally, Section 5 summarizes our findings, draws conclusions, and highlights potential avenues for future research.

## 2 Literature review

In previous studies, questionnaire scales were commonly employed as a means of assessing the level of safety climate in the construction industry. The assessment criteria from the questionnaire scale consisted of multiple factors (Table 1). Some scholars also focused on different safety climate questionnaire factors with the aim of achieving a more precise measurement (Table 1).

**Table 1:** Safety climate factors in construction industry.

Author (year)	Factors	Topic
Choudhry Rafiq et al. (13)	Management commitment, Employee involvement, Inappropriate safety procedure, Work practices	Assess the safety climate of a Hong Kong construction company
Chen et al. (4)	Management commitment to safety, Supervisor safety perception, Coworker safety perception, Reporting, Learning, Anticipation, Awareness	A resilience safety climate model
Kim et al. (38)	Safety commitment, Subcontractor involvement, Safety incentives, and Safety accountability	The interaction effects of safety management and the causes of safety climate on safety performance.
Lestari et al. (14)	Management commitment, Communication, Training, Personal accountability, Rules and procedures, Supportive environment	Assess safety climate and develop a framework
Loosemore et al. (11)	Management commitment, Communication, Rules, Procedures, Supportive environment, Personal accountability, Training	A comparative safety climate of construction operatives and managers.
He et al. (39)	Management commitment, Supervisor perception, Coworker perception, Safety knowledge, Work pressure, Role overload	Comparison between safety climate, safety behaviors, and safety outcomes in two social groups
Chen et al. (40)	Management safety commitment, Supervisor safety role, Co-worker’s role, Safety communication, Safety rules, Procedures, Worker safety involvement, Safety training and competency	A review of safety climate factors in construction industry

Based on the aforementioned literature review, it becomes evident that evaluating safety climate is a multicriteria problem. When variables with multiple demographic attributes are involved, assessing safety climate becomes a complex multi-variables and multi-criteria problem. MCDM methods are commonly used to solve multi-criteria and multi-variables problems. In the past few years, the research of safety climate using MCDM methods gradually increased. For example, using the MCDM methods to rank and compare the effects of safety climate factors and psychosocial safety climate on employee safety performance (17), or using the AHP method to analyze the weights of safety climate evaluation factors (18).

Commonly used MCDM methods include TOPSIS, VIKOR, COPRAS and some improved method. For example, Tang et al. (19) used the grey correlation coefficient to enhance the TOPSIS method for urban sustainability evaluation, specifically addressing the uncertainty existing in the process of evaluation. Similarly, Zeng et al. (20) applied the entropy method to calculate weights of evaluation criteria in the COPRAS method for logistics level evaluation, addressing the need for reasonable weights to obtain accurate results. Additionally, Zhang et al. (21) incorporated prospect theory to improve the VIKOR method by considering decision-makers’ perceived value, resulting in a combination of subjective and objective evaluation for black-start schemes evaluation. Scholars have also proposed new decision matrices to address the scope of applicability of MCDM, such as the new TOPSIS method based on interval data by Jahanshahloo et al. (22), COPRAS-G method based on grey theory by Zavadskas et al. (23) and the extended VIKOR method based on interval number by Sayadi et al. (24). However, the construction of decision matrices in MCDM using real numbers, interval numbers, fuzzy numbers, and random functions fails to fully capture the distribution characteristics and fuzzy nature of the sample data. As a result, Zhang et al. (25) proposed a hybrid method based on cloud model and TOPSIS for SaaS services evaluation, which considers the numerical features of cloud model to construct decision matrices and utilizes the Euclidean distance between clouds in TOPSIS method. Ramakrishnan and Chakraborty (26) proposed a Cloud TOPSIS approach that employs cloud model to construct decision matrices and incorporates the difference between clouds in TOPSIS. It is important to note that the calculation process of above two methods is relatively complex, thus highlighting the need for a method that is both computationally simple and capable of capturing the distribution characteristics of sample data.

Furthermore, when employing MCDM methods, it is crucial to determine the weights of the evaluation criteria. Salabun et al. (27) emphasized that different methods in MCDM utilize different criteria weights, leading to substantial variations in the obtained results, underscoring the significance of accurate criteria weights. The utilization of machine learning for deriving evaluation criteria weights is likely to yield more precise outcomes. Wang et al. (28) employed the importance of variables in random forest based on Gini value, combined with a weighted linear combination, to assess landslide susceptibility. They found that this method was more accurate compared to the entropy method and AHP. Similarly, Gao et al. (29) used the importance of variables in a neural network based on the MIV algorithm to determine the weights of factors influencing the size of a construction site. They observed that this method was more objective compared to AHP. Considering safety climate is a perception, the weights of most evaluation indicators are subject to change with the development of the evaluation object and the evolving understanding of safety. In this regard, the integration of machine learning techniques, such as neural networks, random forests, and other methods, could be considered to establish a learning mechanism for weights, enabling adaptation to changing evaluation requirements.

## 3 Methodology

**Figure 1:**
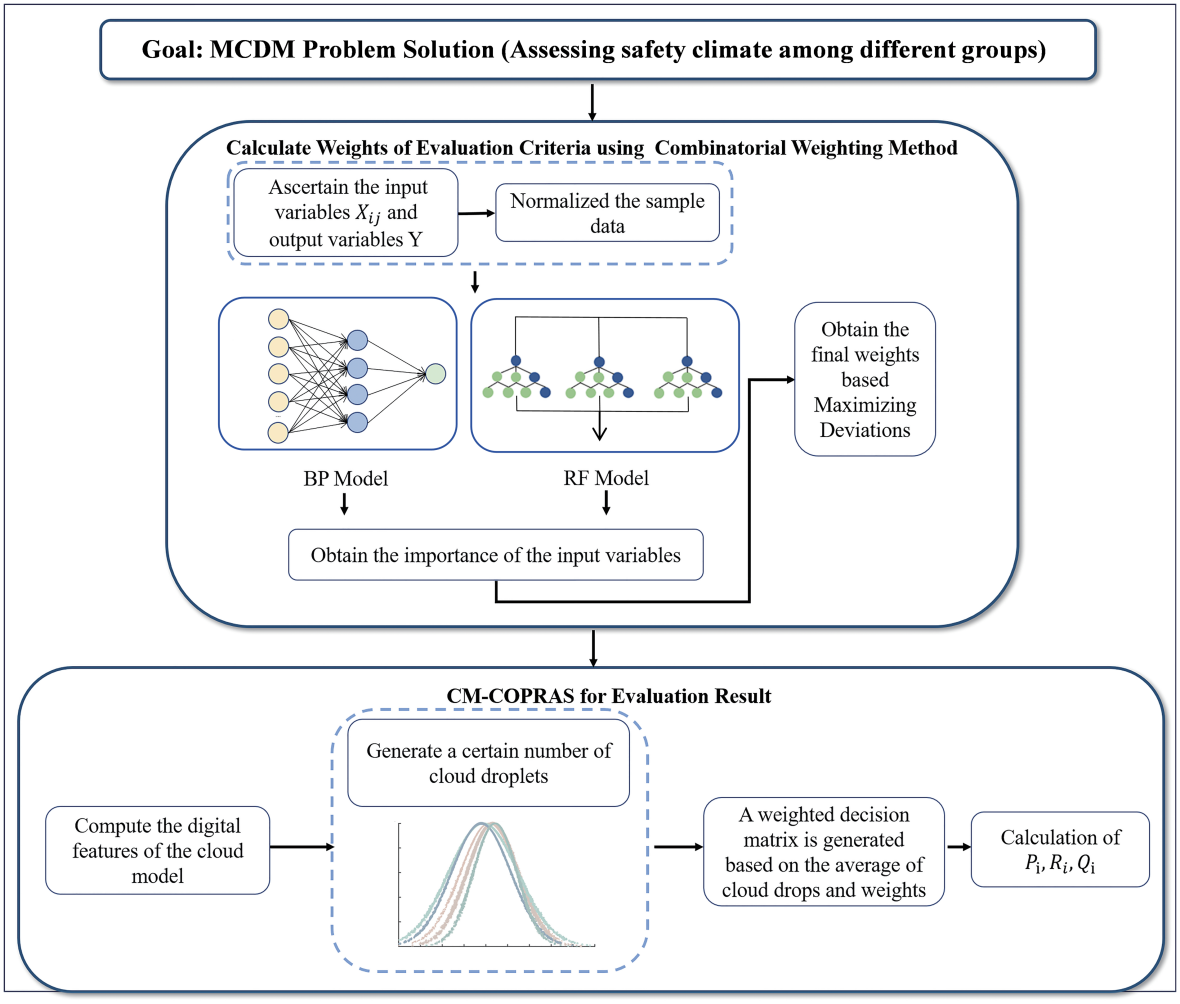
Research methodology flow chart.

Figure 1 presents a comprehensive graphical representation of the overall framework for this hybrid MCDM method, offering a visual illustration of its application. The following text offers an exhaustive exposition of the intricate steps entailed within this method.

### 3.1 Combined weighting method

#### 3.1.1 BP neural network weighting method

Compared with the MIV algorithm, the Garson’s algorithm (30) provides a simpler approach to calculate the importance of input variables in the neural network model. The BP neural network, based on the BP algorithm, is a forward multilayer neural network that employs Scaled Conjugate Gradient algorithm to adjust the coefficients in reverse during the training process, leading to more accurate predictions. In this study, we established the BP neural network model and utilized the Garson’s algorithm to calculate the weights of the evaluation indexes. The calculation steps are as follows:

Step 1: Model Construction.

Determine the number of neurons for the input layer, denoted as *m*, and the output layer, denoted as *n*, based on the input variable $X_{i}$ of sample data in evaluation criteria and the output variable $Y_{k}$ , where i =1, 2, …, *m* and k = 1, 2, …, n .

Calculate the number of neurons for the hidden layer, denoted as *j*, using the following formula:


(1) $$j=\sqrt{m+n}+a$$

where *m* and *n* represent the number of neurons in the input and output layers, respectively, and a is a constant ranging from 1 to 10.

Step 2: Model Training.

To train the neural network, the following formulas are used to calculate the output value of the hidden and output layers, respectively: the output value of the hidden layer, denoted as $Z_{j}$ , is calculated using the formula:


(2) $$Z_{j}=f\left(X_{j}\right)=f\left(b_{j}+\overset{m}{\underset{i=1}{\sum }}v_{ij}x_{i}\right)=\frac{1}{1+\exp\left(-X_{j}\right)}$$

where $v_{ij}$ represents the weight connecting the *i-th* input layer neuron to the *j-th* hidden layer neuron, $b_{j}$ is the threshold of the *j-th* hidden layer neuron, and f is the activation function used to introduce non-linearity to the model.

To calculate the output value of the output layer, denoted as ${\hat{Y}}_{k}$ , use the following equation:


(3) $${\hat{Y}}_{k}=f\left(Z_{k}\right)=f\left(b_{l}+\overset{k}{\underset{j=1}{\sum }}w_{jk}Z_{j}\right)=\frac{1}{1+\exp\left(-Z_{k}\right)}$$

where $w_{jk}$ represents the weight connecting the *j-th* hidden layer neuron to the *k-th* output layer neuron, $b_{k}$ is the threshold of the *k-th* output layer neuron f is the activation function used to introduce non-linearity to the model.

By adjusting the weights and thresholds during training by Scaled Conjugate Gradient algorithm, the neural network can learn to predict the output values for a given set of input values.

Step 3: Obtain the importance of the input variables.

Once the error between the true values and the predicted values, represented by *E*, has been reduced to an acceptable level, the Garson’s algorithm can be utilized to calculate the weights of the variables in evaluation criteria, which are represented by $I_{j}$ . The error function is:


(4) $$E=\frac{1}{2}\overset{n}{\underset{k=1}{\sum }}{\left(Y_{k}-{\hat{Y}}_{k}\right)}^{2}$$

and the Garson’s function is.


(5) $$I_{j}=\frac{\sum_{j=1}^{k}\left(\frac{\left|v_{ij}\times \omega_{ij}\right|}{\sum_{i=1}^{m}\left|v_{ij}\times \omega_{ij}\right|}\right)}{\sum_{i=1}^{m}\sum_{j=1}^{k}\left(\frac{\left|v_{ij}\times \omega_{ij}\right|}{\sum_{i=1}^{m}\left|v_{ij}\times \omega_{ij}\right|}\right)}$$

where $I_{j}$ is the importance of *j-th* input variable.

#### 3.1.2 RF weighting method

In Random Forest, the input variable importance obtained from out-of-bag (*oob*) error method is considered to be more accurate compared to the GINI value method (31). Therefore, in this paper, we chose to use *oob* error method to calculate the weights of evaluation criteria. The calculation steps are as follows:

Step 1: Model Construction.

Ascertain the input variables $X_{ij}$ of sample data in evaluation criteria and output variables Y . Here, i ranges from 1 to *m*, and *j* ranges from 1 to *n*. The value of *m* signifies the total number of samples, while *n* represents the quantity of input variables.

Step 2: Model Training.

K samples are selected from the input variables $X_{ij}$ through the utilization of the Bootstrap self-sampling technique, aiming to construct a collection of K Regression Trees denoted as ${\left\{T\left(x\right)\right\}}_{1}^{k}$ for training purposes. The samples that remain unselected during this process are commonly referred to as out-of-bag ( oob ) samples. The RF regression predictor estimates the value $f_{rf}^{\hat{i}}$ by computing the sum of predictions from each tree and dividing it by the total number of trees ( k ), as expressed by the equation:


(6) $$f_{rf}^{\hat{i}}=\frac{1}{k}\overset{k}{\underset{i=1}{\sum }}T\left(x\right)$$

Step 3: Obtain the importance of the input variables.

The importance of each feature variable $I_{j}$ is determined by evaluating the formula:


(7) $$I_{j}=\frac{\sum \left(e_{2}-e_{1}\right)}{k}$$

where $e_{1}$ represents the prediction error based on the out-of-bag ( oob ) data, and $e_{2}$ corresponds to the prediction error based on the modified out-of-bag data ( $oob^{\prime }$ ) for each tree. The modified out-of-bag data ( $oob^{\prime }$ ) is generated by introducing random variations and incorporating noise interference into the original out-of-bag ( oob ) samples.

#### 3.1.3 Maximizing deviations

Liu and Hu (32) utilized a combined weighting method based Maximizing Deviations (MD) to determine attribute weights in the multi-attribute decision problems. This method aims to synthesize the distinctive aspects of two weighting methods. The following steps outline the process of combining the weightings using the MD.

Step 1: The maximum sum of squared deviations of the samples can be calculated using the formula:


(8) $$\max Z=\overset{n}{\underset{j=1}{\sum }}\overset{m}{\underset{i=1}{\sum }}{\left(r_{ij}-{\bar{r}}_{ij}\right)}^{2}\cdot \omega_{ij}=\overset{n}{\underset{j=1}{\sum }}\overset{m}{\underset{i=1}{\sum }}{\left(r_{ij}-{\bar{r}}_{ij}\right)}^{2}\left(\begin{array}{l} \alpha \omega_{j1}+\\ \beta \omega_{j2} \end{array}\right)$$

In this formula, $r_{ij}$ represents the sample data, $\omega_{j}$ is the combination weight, $\omega_{j1}$ is the *j-th* criterion weight assigned by the BP weighting method, $\omega_{j2}$ is the *j-th* criterion weight assigned by the RF weighting method, ${\bar{r}}_{ij}$ denotes the average value of $r_{ij}$ , α and β are coefficients, and j ranges from 1 to *n* while i ranges from 1 to *m*. The constraint conditions are as follows:


(9) $$\{\alpha^{2}+\beta^{2}=1,\alpha >0,\beta >0$$

These conditions ensure the validity of the combination weighting process.

Step 2: The Lagrange function for solving α and β is constructed as follows:


(10) $$L\left(\alpha ,\beta \right)=\overset{n}{\underset{j=1}{\sum }}\overset{m}{\underset{i=1}{\sum }}{\left(r_{ij}-{\bar{r}}_{ij}\right)}^{2}\cdot \left(\alpha \mu_{j}+\beta v_{j}\right)+\lambda \left(\alpha^{2}+\beta^{2}-1\right)$$

Here, λ represents the Lagrange multiplier. To find the optimal values of α and β , we take the partial derivatives:


(11) $$\{\begin{array}{c} \overset{n}{\underset{j=1}{\sum }}\overset{m}{\underset{i=1}{\sum }}{\left(r_{ij}-{\bar{r}}_{ij}\right)}^{2}\cdot u_{j}+2\lambda \alpha =0\\ \overset{n}{\underset{j=1}{\sum }}\overset{m}{\underset{i=1}{\sum }}{\left(r_{ij}-{\bar{r}}_{ij}\right)}^{2}\cdot v_{j}+2\lambda \beta =0 \end{array}$$

In addition, considering the constraint $\alpha^{2}+\beta^{2}=1$ , we can express α and β as follows:


(12) $$\alpha =\frac{1}{\sqrt{1+\frac{\overset{n}{\underset{j=1}{\sum }}\overset{m}{\underset{i=1}{\sum }}{\left(r_{ij}-{\bar{r}}_{ij}\right)}^{2}\cdot u_{j}}{\overset{n}{\underset{j=1}{\sum }}\overset{m}{\underset{i=1}{\sum }}{\left(r_{ij}-{\bar{r}}_{ij}\right)}^{2}\cdot v_{j}}}}$$

(13) $$\beta =\frac{1}{\sqrt{1+\frac{\overset{n}{\underset{j=1}{\sum }}\overset{m}{\underset{i=1}{\sum }}{\left(r_{ij}-{\bar{r}}_{ij}\right)}^{2}\cdot v_{j}}{\overset{n}{\underset{j=1}{\sum }}\overset{m}{\underset{i=1}{\sum }}{\left(r_{ij}-{\bar{r}}_{ij}\right)}^{2}\cdot u_{j}}}}$$

Step 3: The final weights, denoted as $\omega_{j}$ , are calculated using the formula:


(14) $$\omega_{j}=\alpha^{\prime }u_{j}+\beta^{\prime }v_{j}$$

Here, $\alpha^{\prime }$ and $\beta^{\prime }$ are the normalized coefficients obtained from α and β , respectively.

### 3.2 CM-COPRAS method

#### 3.2.1 Cloud model

The cloud model, originally proposed by Li et al. (33), can be described as follows: Let U represent the universe of discourse consisting of exact values, and C be a qualitative concept within U . A quantitative value $x_{i}$ belongs to U and is a one-time random instantiation of concept C . If $x_{i}$ follows a normal distribution $N\sim \left(Ex^{2},Enn^{2}\right)$ and Enn follows a normal distribution $N\sim \left(En,He^{2}\right)$ , the membership degree of $x_{i}$ pertaining to the concept A can be calculated as:


(15) $$\left(x_{i}\right)=\exp\left(-\frac{{\left(x_{i}-Ex\right)}^{2}}{2Enn^{2}}\right)$$

The distribution of $x_{i}$ within *U* is referred to as a cloud, and each instance ${\hat{x}}_{i}$ is called a cloud drop. In the cloud model, cloud is composed of numerous cloud droplets ${\hat{x}}_{i}$ .

The numerical characteristics of the cloud model are represented by three cloud parameters Ex , En , and He , where Ex responds to the average value in all samples, $E_{n}$ responds to the fuzziness and concentration in all samples, and He responds to the stability of the entropy. The MBCT-SR algorithm (34) is used to calculate (Ex,En,He) , which steps are as follows:

Step 1: Input *m* sample $x_{k}$ (*k* = 1, 2, 3…, *m*), where *k* denotes the sample sequence.

Step 2: Calculate the sample average value to obtain an estimate of the expected Ex :


(16) $$Ex=\frac{1}{m}\overset{m}{\underset{k=1}{\sum }}x_{k}$$

Step 3: From the m original samples, randomly and repeatedly draw c samples to form a d-sample set. The variance of the *l-th* group sample, denoted as $t_{l}^{2}$ , is calculated as follows:


(17) $$t_{l}^{2}=\frac{1}{c-1}\overset{c}{\underset{r=1}{\sum }}{\left(x_{lr}-Ex_{l}\right)}^{2};l=1,2\ldots ,c$$

where $Ex_{l}$ is sample average value within group.

Step 4: Calculate the estimates of $En^{2}$ and $He^{2}$ :


(18) $${\left(ET\right)}^{2}=\frac{1}{d}\overset{d}{\underset{l=1}{\sum }}t_{l}^{2}$$

(19) $${\left(DT\right)}^{2}=\frac{1}{d-1}\overset{d}{\underset{l=1}{\sum }}{\left(t_{l}^{2}-{\left(ET\right)}^{2}\right)}^{2}$$

(20) $$\{\begin{array}{c} En^{2}=\frac{1}{2}\sqrt{4{\left({\left(ET\right)}^{2}\right)}^{2}-2{\left(DT\right)}^{2}}\\ He^{2}={\left(ET\right)}^{2}-En^{2} \end{array}$$

#### 3.2.2 CM-COPRAS

The CM-COPRAS model is a combination of cloud model and COPRAS with the following steps.

Step 1: The numerical characteristics of the cloud model (Ex,En,He) are calculated to obtain the cloud decision matrix $C_{ij}$ .


(21) $$C_{ij}=\begin{array}{c} \left(Ex_{11},En_{11,}He_{11}\right)\;\;\ldots \;\;\left(Ex_{1n},En_{1n},He_{1n}\right)\\ \begin{array}{ccc} \left(Ex_{21},En_{21,}He_{21}\right) & \ldots & \left(Ex_{2n},En_{2n},He_{2n}\right) \end{array}\\ \begin{array}{c} \begin{array}{ccc} \vdots & \ddots & \vdots \end{array}\\ \begin{array}{ccc} \left(Ex_{m1},En_{m1,}He_{m1}\right) & \ldots & \left(Ex_{mn},En_{mn},He_{mn}\right) \end{array} \end{array} \end{array}$$

*m* is the number of demographic variables in group and j is the number of evaluation criteria.

Step 2: Generation of a random number Hnn with En as the expected value and He as the variance. Then, generate cloud droplet ${\hat{x}}_{i}$ with Ex as the expectation and Hnn as the variance. Repeat this arrangement to generate *n* cloud droplets. The average value of the cloud droplets denoted as $r_{ij}$ , is used to construct the decision matrix $R_{ij}$ , which is formulated as:


(22) $${\hat{x}}_{i}=rand\left(1\right)\cdot \left[rand\left(1\right)\cdot He_{ij}+En_{ij}\right]+Ex_{ij}$$

(23) $$r_{ij}=\frac{1}{n}\overset{n}{\underset{k=1}{\sum }}{\hat{x}}_{i}$$

(24) $$R_{ij}=\left(\begin{array}{ccc} r_{11} & \cdots & r_{1n}\\ \vdots & \ddots & \vdots \\ r_{m1} & \cdots & r_{mn} \end{array}\right)$$

Step 3: Normalization of Decision Matrix $d_{ij}$ . The formulation of normalization is:


(25) $$d_{ij}=\frac{r_{ij}}{\overset{m}{\underset{i=1}{\sum }}r_{ij}}$$

The normalized decision matrix is as follows:


(26) $$d_{ij}=\left(\begin{array}{ccc} d_{11} & \ldots & d_{1n}\\ \vdots & \ddots & \vdots \\ d_{m1} & \cdots & d_{mn} \end{array}\right)$$

Step 4: Formation of Weighted Matrix.

The weighted matrix, denoted as ${\hat{d}}_{ij}$ , is obtained as follows.


(27) $${\hat{d}}_{ij}=\omega_{ij}d_{ij}=\left(\begin{array}{ccc} {\hat{d}}_{11} & \ldots & {\hat{d}}_{1n}\\ \vdots & \ddots & \vdots \\ {\hat{d}}_{m1} & \cdots & {\hat{d}}_{mn} \end{array}\right)$$

where $\omega_{j}$ is the weight of *j-th* evaluation criterion.

Step 5: Calculation of $P_{i}$ , $R_{i}$ and $Q_{i}$ .

$P_{i}$ is identified by the summation over the k beneficial criteria and $R_{i}$ is identified by the summation over the n-k non-beneficial criteria. The priority of ranks in safety climate among different groups can be calculated from significance $Q_{i}$ . The formulation of $P_{i}$ , $R_{i}$ and $Q_{i}$ is:


(28) $$P_{i}=\overset{k}{\underset{j=1}{\sum }}{\hat{d}}_{ij}$$

(29) $$R_{i}=\overset{n}{\underset{j=k+1}{\sum }}{\hat{d}}_{ij}$$

(30) $$Q_{i}=P_{i}+\frac{R_{\min}\sum_{i=1}^{m}R_{i}}{R_{i}\sum_{i=1}^{m}\frac{R_{\min}}{R_{i}}}=P_{i}+\frac{\sum_{i=1}^{m}R_{i}}{R_{i}\sum_{i=1}^{m}\frac{1}{R_{i}}}$$

(31) $$R_{\min}=\underset{1\leq i\leq m}{\min}\left\{R_{j}\right\}$$

Step 6: Calculation of utility degree $U_{i}$ from $Q_{i}$ . The formulation of $U_{i}$ is:


(32) $$U_{i}=\frac{Q_{i}}{Q_{\max}}$$

(33) $$Q_{\max}=\underset{1\leq i\leq m}{\max}Q_{i}$$

## 4 Results

This study was conducted for a road construction company in China. The evaluation criteria were selected from a safety climate questionnaire scale, and the sample data consisted of questionnaire responses collected from roadway construction workers. The collected data was then used in the hybrid MCDM approach proposed in this study to assess the safety climate of different groups.

**Figure 2:**
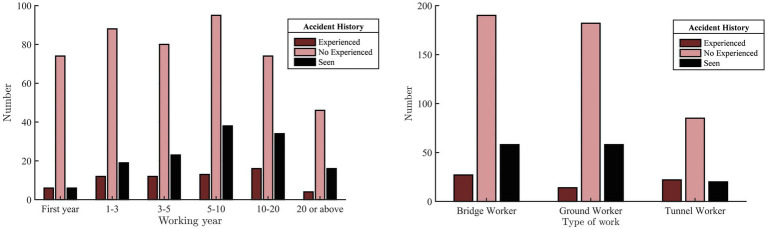
Number of people with different accident experiences.

The safety climate questionnaire scale employed in this case study consisted of 16 items focusing on graphic inquiries and a five-point Likert-type scale ranging from 1 (strongly disagree) to 5 (strongly agree) with 52 items to collect workers’ responses regarding various aspects of safety climate. A total of 800 questionnaires were distributed among construction workers at CITIC Yunnan Branch of Chu Da Expressway, resulting in the collection of 710 questionnaires. A data cleansing process was then conducted to eliminate duplicate answers, missing responses, abnormal values, answers that violated specific rules, and inconsistent answers. The final dataset comprised 656 workers (Table 2). Among the workers, the age group with the highest representation was 41–50 years (35.37%), while the age group with the lowest representation was 18–30 years (17.7%). Furthermore, a majority of workers had a junior high school education (51.07%). As depicted in Figure 2, the group with a work experience ranging from 10 to 20 years witnessed the highest number of accidents, while the group with over 20 years of work experience experienced the lowest number of accidents. Among the surveyed workers, the bridge worker group had the highest number of accidents, whereas the ground worker group had the lowest number of accidents.

**Table 2:** Sample statistics.

Variables	Category	Number of samples	Proportion (%)
Gender	Male	612	93.29
	Female	44	6.71
Age (years)	18–30	116	17.7
	31–40	173	26.37
	41–50	232	35.37
	51 or older	130	19.82
Education	Primary or no	221	33.69
	Junior high school	335	51.07
	High school and above	100	14.18

An exploratory factor analysis (EFA) was conducted using SPSS 25 to identify the underlying factors contributing to the safety climate evaluation criteria. The Kaiser Meyer-Olkin (KMO) measure of sampling adequacy yielded a value of 0.862, indicating the suitability of the data for factor analysis (35). Four factors were extracted based on eigenvalues greater than 1, and all items exhibited factor loadings exceeding 0.4 on their respective factors. These factors were labelled as follows: safety communication (F1), safety management (F2), safety attitude (F3), and safety awareness (F4). F1 comprised 10 items, F2 comprised 7 items, F3 comprised 5 items, and F4 comprised 5 items. Confirmatory factor analysis (CFA) was subsequently conducted in Amos 27, and the model fit indices met the required criteria (RMSEA = 0,038, $\chi^{2}/df$ = 1,966, GFI = 0,935, AFGI = 0,923, IFI = 0,903, TLI = 0,892, CFI = 0,902), indicating an acceptable fit (36). The overall Cronbach’s alpha coefficient was calculated to be 0.93, with alpha values of 0.78, 0.71, 0.69, and 0.66 for safety communication, safety management, safety attitude, and safety awareness, respectively, demonstrating acceptable reliability (37). Based on the results of above, the safety climate evaluation criteria consisted of 27 items, as outlined in Appendix_1.

**Figure 3:**
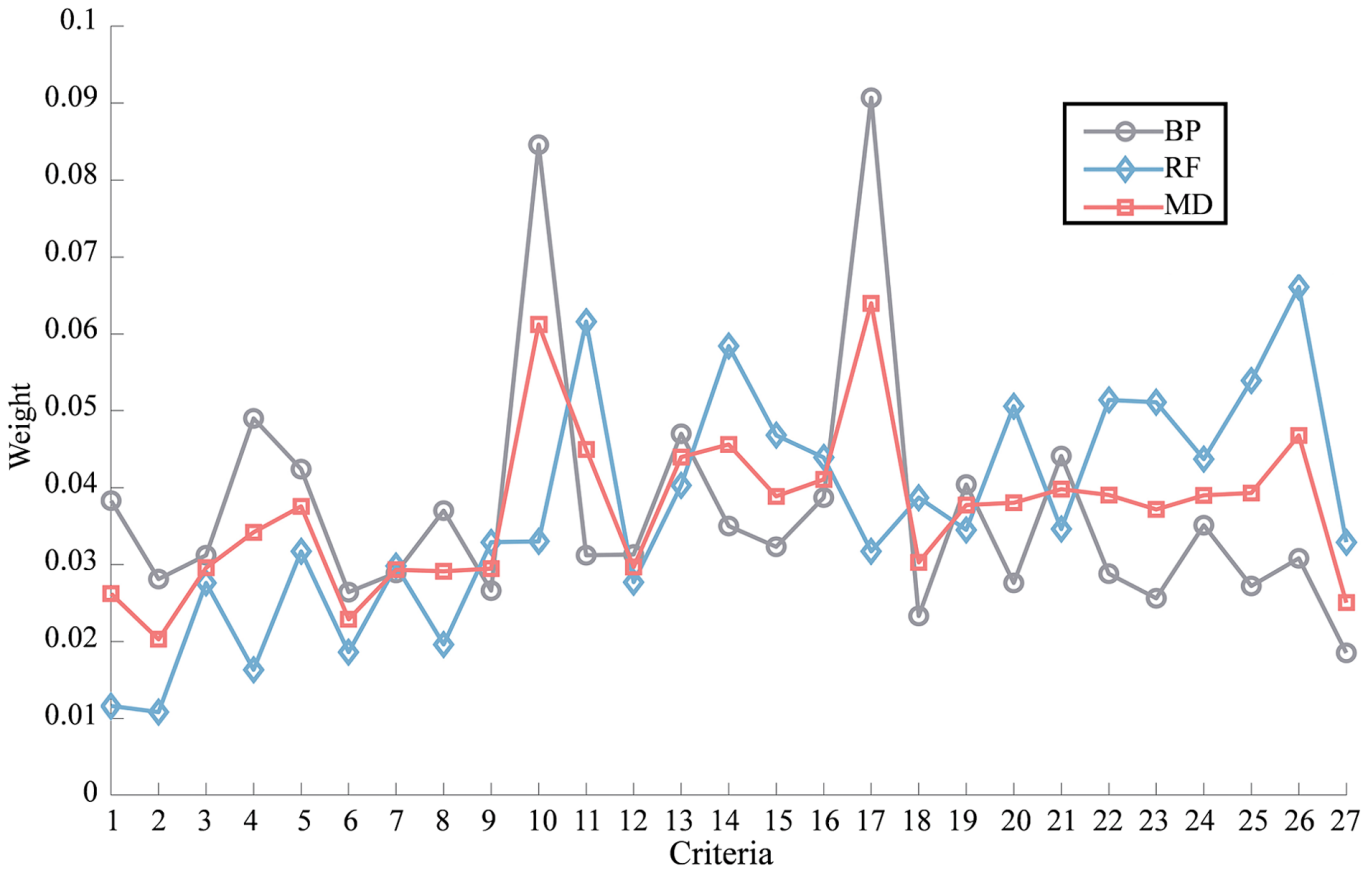
Weights of evaluation criteria.

The 656 samples data under 27 evaluation criteria were imported into the BP neural network model and normalized by min-max normalization as input variables, and the factor analysis result was normalized by min-max normalization as output variable. The absolute value of Pearson correlation coefficients between the normalized input and output variables were found to be less than 0.6 and statistically significant, indicating the presence of a non-linear relationship between these variables. So, the neural network architecture consisted of an input layer with 27 neurons, an output layer with a single neuron, and a hidden layer comprising 7 neurons. The number of neurons in the hidden layer was calculated using Equation 1. The dataset was randomly divided into a 70% training set and a 30% test set. After 73 iterations with Equations 2–4 and Scaled Conjugate Gradient algorithm, the training of the BP neural network was completed, resulting in a final prediction error of 2.65 × 10^–5^ for the test set samples. The weights of all safety climate criteria were determined by Equation 5, and the results were depicted in Figure 3.

The same input and output variables were used in the RF method. Similarly, the dataset was randomly divided into a 70% training set and a 30% test set. After training, the error on the test set was found to be 0.58 × 10^−2^ using Equation 6. The weights of all safety climate criteria were determined using Equation 7, and the results were illustrated in Figure 3.

**Figure 4:**
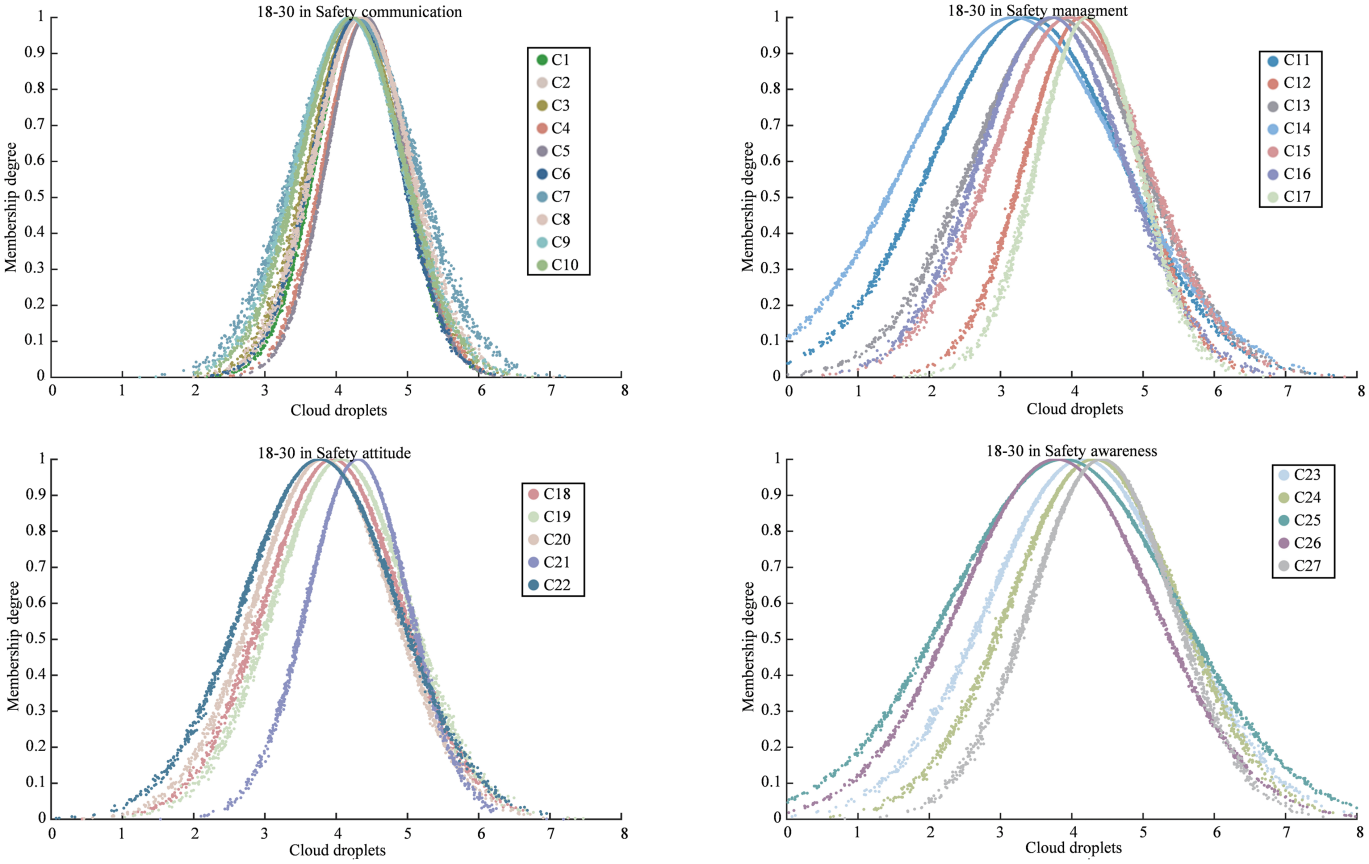
The cloud model for the safety climate criteria (C1 to C27) within the 18–30 in age group.

**Figure 5:**
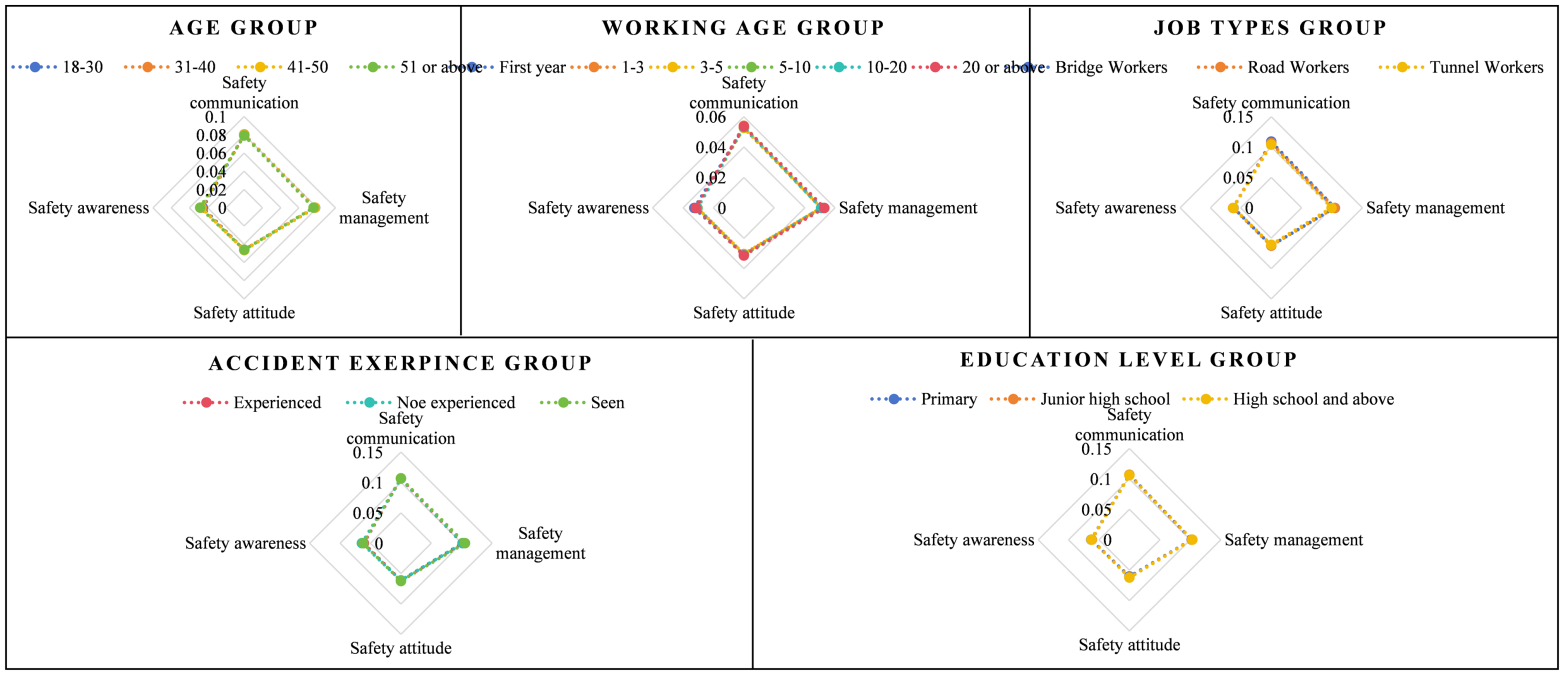
The calculation results of four factors for different groups.

After obtaining the criteria weights using both the BP weighting method and the RF weighting method, the final weights were calculated by employing the combination weight of MD using Equations 8–12. The results of these calculations were illustrated in Figure 3. Calculate the numerical characteristics (Ex,En,He) of the cloud model in safety climate criteria for various sample groups to obtain the cloud decision matrix using Equations 16–21, as presented in Table 3. Cloud droplets are then generated based on Equation 22, with a total of 2,000 cloud droplets considered in this study. For better understanding, Figure 4 provides an example of the cloud droplets for the four safety climate criteria within the 18–30 group. It demonstrates how the cloud model can visually represent the characteristics and relationships of the criteria. The average values of cloud droplets $r_{ij}$ are calculated using Equation 23 and subsequently used to construct the decision matrix using Equation 24, as presented in Table 3. The decision matrix is normalized and combined with weights to generate the weighted decision matrix using Equations 25–27, as depicted in Table 3. The significance evaluation $Q_{i}$ and utility degree evaluation $U_{i}$ are then performed using Equations 28–30. Criteria related to safety communication, safety management, and safety attitude are considered beneficial for $P_{i}$ , while those associated with safety awareness are deemed non-beneficial for $R_{i}$ . The calculation results of safety climate for different groups are summarized in Table 4. In same way, the calculation results of four factors for different groups are summarized in Figure 5.

**Table 3:** Numerical characteristics of the cloud model and decision matrix.

	C1	C2	…C27
	$\left(Ex,En,He\right)r_{ij}$	$\left(Ex,En,He\right)r_{ij}$	$\left(Ex,En,He\right)r_{ij}$
Age group			
18–30	(4.3141, 0.6042, 0.0033)4.3461	(4.3058, 0.6269, 0.0037)4.3650	(4.4215, 0.9945, 0.0090)4.3529
31–40	(4.3236, 0.7603, 0.0238)4.3348	(4.3237, 0.7450, 0.0239)4.3192	(4.1272, 1.2266, 0.0173)4.0998
41–50	(4.3793, 0.6875, 0.0238)4.3831	(4.3534, 0.6460, 0.0159)4.3158	(4.1207, 1.2843, 0.0179)4.1430
51 or older	(4.2846, 0.8396, 0.0444)4.2269	(4.3538, 0.6347, 0.0322)4.3548	(4.1769, 1.1521, 0.0418)4.1660
Working year group			
First year	(4.3140, 0.6346, 0.0123)4.3189	(4.2674, 0.6764, 0.0169)4.2930	(4.1047, 1.3450, 0.0181)4.0951
1–3	(4.3613, 0.6464, 0.0220)4.3436	(4.3697, 0.6905, 0.0191)4.3820	(4.2521, 1.1734, 0.0208)4.2449
3–5	(4.3565, 0.6202, 0.0121)4.3596	(4.3217, 0.7077, 0.0202)4.3194	(4.1043, 1.1735, 0.0235)4.1125
5–10	(4.3630, 0.7314, 0.0171)4.3800	(4.3630, 0.5689, 0.0064)4.3545	(4.1712, 1.2140, 0.0267)4.1556
10–20	(4.3468, 0.8491, 0.0227)4.3455	(4.3306, 0.7277, 0.0157)4.3167	(4.2501, 1.1933, 0.0261)4.2026
20 or over	(4.2576, 0.8577, 0.0231)4.2587	(4.3485, 0.6144, 0.0073)4.3673	(4.2576, 1.0297, 0.0222)4.2588
Accident experience group			
Experienced	(4.2698, 0.7612, 0.0208)4.2654	(4.3651, 0.6487, 0.0137)4.3697	(4.3968, 1.1252, 0.0232)4.3799
Not experienced	(4.3020, 0.7494, 0.0208)4.3328	(4.3217, 0.6842, 0.0201)4.3091	(4.1838, 1.1925, 0.0154)4.1818
Seen	(4.5074, 0.5952, 0.0129)4.4926	(4.3750, 0.6268, 0.0118)4.3622	(4.1103, 1.2399, 0.0181)4.0289
Job types group			
Bridge Workers	(4.4255, 0.6642, 0.0228)4.4469	(4.4109, 0.6179, 0.0108)4.3820	(4.2655, 1.1465, 0.0192)4.2688
Road Workers	(4.2717, 0.8003, 0.0230)4.2727	(4.3228, 0.7123, 0.0276)4.3169	(4.0591, 1.2781, 0.0230)4.0189
Tunnel Workers	(4.2992, 0.6776, 0.0128)4.3312	(4.2047, 0.6521, 0.0128)4.2111	(4.2835, 1.1165, 0.0216)4.3028
Education level group			
Primary	(4.3756, 0.6846, 0.0136)4.4037	(4.3756, 0.6649, 0.0234)4.4087	(4.2443, 1.1401, 0.0294)4.2228
Junior high school	(4.3433, 0.7481, 0.0194)4.3213	(4.3224, 0.6760, 0.0190)4.3585	(4.1194, 1.2355, 0.0191)4.2111
High school and above	(4.2600, 0.7245, 0.0188)4.2880	(4.3000, 0.6587, 0.0102)4.3141	(4.3000, 1.2030, 0.0246)4.3119

**Table 4:** Evaluation results of CM-COPRAS and other method.

Methods	CM-COPRAS			CLOUD-TOPSIS1		CLOUD-TOPSIS2	
	*Q_i_*	*U_i_*	Rank	*R_i_*	Rank	*R_i_*	Rank
Age group							
18–30	0.2497	0.9955	3	0.4559	3	0.4571	3
31–40	0.2498	0.9959	2	0.5257	2	0.4993	2
41–50	0.2508	1	1	0.5439	1	0.5545	1
51 or older	0.2496	0.9953	4	0.4371	4	0.3906	4
Working age group							
First year	0.1677	0.9889	2	0.5203	2	0.4501	4
1–3	0.1661	0.9792	4	0.4604	4	0.5278	2
3–5	0.1643	0.9687	6	0.3103	6	0.3271	6
5–10	0.1665	0.9819	3	0.4853	3	0.4916	3
10–20	0.1657	0.9770	5	0.3425	5	0.4142	5
20 or over	0.1697	1	1	0.7316	1	0.6696	1
Accident experience group							
Experienced	0.3301	0.9812	3	0.3913	3	0.4022	3
Not experienced	0.3333	0.9907	2	0.4714	2	0.454	2
Seen	0.3365	1	1	0.5724	1	0.6193	1
Job types group							
Bridge workers	0.3380	1	1	0.7839	1	0.7966	1
Road workers	0.3356	0.9930	2	0.6608	2	0.6076	2
Tunnel workers	0.3264	0.9658	3	0.0936	3	0.1004	3
Education level group							
Primary	0.3324	0.9944	3	0.3732	3	0.4295	3
Junior high school	0.3333	0.9973	2	0.6258	1	0.651	1
High school and above	0.3342	1	1	0.5447	2	0.472	2

One CLOUD-TOPSIS by Long-chang et al. (42); 2 CLOUD TOPSIS by Ramakrishnan and Chakraborty (26); where λ_1_ = 1, λ_2_ = λ_3_ = 0.

## 5 Discussions

In order to validate the accuracy of the proposed model, we compare the proposed model with existing safety climate assessment methods. The current methods of safety climate assessment are mainly questionnaires and MCDM, in which the common method in MCDM is TOPSIS. Our study is based on questionnaire scales from existing studies to construct evaluation indicators for safety management, safety communication, safety attitude, and safety awareness. Therefore, the study compares the proposed model with CLOUD-TOPSIS, and the results calculated based on Cloud-TOPSIS are shown in Table 4.

As shown in Table 4, the results from CM-COPRAS indicate that among the different age groups, workers aged 41–50 demonstrated the highest level of safety climate, with a validity value of 100%. Conversely, workers aged 51 or above had the lowest level of safety climate, with a validity value of 99.5%. Therefore, the ranking of safety climate levels across age groups was as follows: 41–50 > 31–40 > 18–30 > 51 or above. Regarding the working age group, the ranking of safety climate levels was as follows: 20 or over> first year>5–10 > 1–3 > 10–20 > 3–5. In terms of accident experience, the ranking of safety climate levels was as follows: witnessed an accident > did not experience an accident > experienced an accident. Considering different work types, the ranking of safety climate levels was as follows: bridge worker > road worker > tunnel worker. In the education group, the ranking of safety climate levels is as follows: high school and above > junior high school > primary school.

Based on the results presented in Figure 5, we can adopt a targeted approach to enhance the safety climate among different groups. Taking the age groups into consideration, workers aged 51 or above scored lower in safety management and safety communication. To address this, it is advisable to offer more comprehensive safety training and education, with a strong emphasis on the significance of safety management, encompassing strict compliance and enforcement of rules and regulations. Moreover, promoting safety communication and encouraging experience sharing within this age group can promote safer behaviors. The group of workers aged 31–40 exhibits low scores in safety attitudes, which can be rectified by providing them with more safety information and case studies. By doing so, they can better grasp the gravity and importance of safety issues, thereby nurturing a heightened sense of responsibility. Similarly, the group of workers aged 18–30 demonstrates low scores in safety awareness, necessitating the reinforcement of their safety consciousness. To achieve this, organizing safety education activities will play a pivotal role in conveying essential safety knowledge and skills. Consequently, they will be better equipped to identify potential hazards and risks, and acquire the appropriate safety practices.

It should be noted that the three models were almost consistent in assessing the safety climate for working age groups, accident experience groups, and work type groups. However, a slight ranking difference between the three methods emerged in the education groups, where the education groups were ranked as High school and above > Junior high school > Primary school. In the method proposed in this paper, while the two CLOUD TOPSIS methods were ranked as Junior high school > High school and above > Primary school. In order to explain the reasons for the differences, the three methods are compared.

CM-TOPSIS1 assesses each weighted cloud ( $\omega_{j}Ex_{ij},\omega_{j}En_{ij},\omega_{j}He_{ij}$ ) by calculating its Euclidean distance from the ideal cloud ( $d_{i}^{+}$ ) and negative ideal ( $d_{i}^{-}$ ), and calculate $R_{i}$ ( $R_{i}=\frac{d_{i}^{+}}{d_{i}^{+}+d_{i}^{-}}$ ), to select the clo ud that is closest to the ideal cloud and furthest from the negative ideal cloud as the best choice. Among which, ideal cloud is (max( $\omega_{j}Ex_{ij}$ ), min( $\omega_{j}En_{ij}$ ), min( $\omega_{j}He_{ij}$ )); Negative cloud is (min( $\omega_{j}Ex_{ij}$ ), max( $\omega_{j}En_{ij}$ ), max( $\omega_{j}He_{ij}$ )); The calculation processes of CM-TOPSIS2 and CM-TOPSIS1 differ significantly. In CM-TOPSIS2, the distance *d* between clouds is not calculated using the Euclidean distance method.


$$\begin{array}{l} d=\lambda_{1}=\frac{\left|Ex_{i}-Ex_{j}\right|}{\max\left(Ex_{i}+3En_{i},Ex_{j}+3En_{j}\right)-\min\left(Ex_{i}-3En_{i},Ex_{j}-3En_{j}\right)}\\ +\lambda_{2}(1-\frac{\min\left(En_{i},En_{j}\right)}{\max\left(En_{i},En_{j}\right)})+\lambda_{3}(1-\frac{\min\left(He_{i},He_{j}\right)}{\max\left(He_{i},He_{j}\right)}),\left(\lambda_{1}+\lambda_{2}+\lambda_{3}=0\right) \end{array}$$

While weighted cloud is ( $\omega_{j}Ex_{ij},\sqrt{{\left(\omega_{j}En_{ij}\right)}^{2}},\sqrt{{\left(\omega_{j}He_{ij}\right)}^{2}}$ ). The rest of the computational steps are consistent with CM-TOPSIS1. Compared with CM-TOPSIS, CM-COPRAS does not need to calculate positive and negative ideal solutions, and the calculation process is simpler. In CM-TOPSIS, if the positive and negative ideal solutions are not set properly, it may lead to errors in the calculation results. CM-COPRAS uses the COPRAS method for ranking and selection, focusing on the assessment of both beneficial and non-beneficial criteria, unlike the CM-TOPSIS methods. A unique aspect of CM-COPRAS is utility degree calculation, which helps determine final rankings and safety climate levels, a process not covered in the other two models. These distinct approaches ensure that the method proposed in this paper achieves a higher degree of accuracy.

Additionally, because TOPSIS ranks alternatives based on their distance from ideal solutions, it is more sensitive to extreme values. The differences in the education group rankings may stem from the influence of a small number of highly educated workers in the sample, which could have skewed the TOPSIS results. The consistency of the computational results of the CM-COPRAS model with the results of the existing research, i.e., that the education level is positively proportional to the safety climate (8) supports the reasonableness and validity of the CM-COPRAS model.

Although CM-COPRAS yielded results consistent with existing literature, the method relies heavily on data distribution due to its use of a cloud model to construct the decision matrix with cloud droplets. Nonetheless, it represents a novel MCDM approach. This method can be employed to assess the safety climate among different groups of construction workers, enabling the development of targeted interventions.

## 6 Conclusion

In assessing the safety climate within the construction industry, it is challenging to compare the levels of safety climate among different groups of construction workers and determine the areas in which certain groups excel over others. This difficulty arises due to the multitude of evaluative indicators and demographic variables involved. To address this issue, we propose a novel MCDM method for assessing the safety climate of different groups.

The CM-COPRAS method proposed in this study effectively addresses the limitations of current assessment methodology. In our approach, we utilize the BP neural network and random forest algorithm to establish a weight learning mechanism for calculating the weights of safety climate evaluation criteria, which ensures the accuracy of weights. Subsequently, the cloud model is employed to construct the decision matrix for different groups under the evaluation criteria. To compare the safety climate of different groups, we utilize the COPRAS method. We substantiate the effectiveness of our proposed method through a case study conducted within an actual construction company. Furthermore, we compare the results with those obtained from other MCDM methods to verify the accuracy of our approach. Notably, our method offers practicality as it can provide targeted measures to address the weaknesses identified in various groups.

The proposed method possesses the following characteristics: (1) simplicity in calculation and ease of operation; (2) logical reasoning supported by a solid mathematical and theoretical foundation; (3) capability to accommodate the distribution characteristics of sample data through cloud model; (4) objective and realistic weights obtained from the calculations, capable of adapting to the evolution and changes in evaluation indexes.

A stream of future research can consider to enhance road construction safety based on the assessment results obtained from our method. One potential direction is to focus on group behavior within construction teams and develop interventions or training programs that target specific areas of improvement identified by our assessment. By addressing group dynamics and promoting a culture of safety within construction teams, it is possible to achieve better safety outcomes on road construction sites. Additionally, future research can investigate the integration of our method with other complementary approaches to further enhance its effectiveness. For example, optimization algorithms can be utilized to optimize safety measures and resource allocation based on the assessment results. By leveraging prediction algorithms, it becomes possible to anticipate potential safety risks or incidents, allowing for proactive interventions and preventive measures.

Moreover, several limitations of this study should be acknowledged. Although the proposed questionnaire factors are based on a large number of existing research cases involving various industry regions and are representative to a certain extent, this study has not validated the cases in other regions and industries, so the generalizability of the proposed methodology needs further practice. Therefore, a potential future direction could be to validate the level of safety climate in other industries and regions based on the methodology proposed in this paper. Additionally, while the methodology is robust, the hybrid approach’s complexity may present implementation challenges without specialized knowledge or training. Future research could build on the method proposed in this paper to develop an operational system specifically designed for frontline workers. This system would enable them to simply collect and input data, with the system automatically calculating the safety climate rankings for the study groups, without requiring them to understand the complex algorithm logic.

## Data Availability

The original contributions presented in the study are included in the article/supplementary material, further inquiries can be directed to the corresponding author.

## Appendix 1

Safety Climate Evaluation Criteria.

**Table:**

Safety Climateactors	Title number	Criteria
Safety communication	C1	I will proactively seek help from my colleagues when I encounter something I don’t know how to do.
	C2	The team leader often instructs me on how to pay attention to safety.
	C3	When there are changes in safety regulations, the team leader or safety officer will promptly inform us.
	C4	If I discover a safety hazard, I will report the situation to the team leader.
	C5	When I don’t follow proper procedures, my colleagues will promptly remind and correct me.
	C6	When I see someone operating incorrectly, I will proactively go and inform them.
	C7	I will proactively communicate safety work experiences with my colleagues, even if management doesn’t emphasize it.
	C8	After work is completed, the team leader or colleagues will remind me to place the used equipment in a safe location.
	C9	I receive very prompt responses and resolutions for the issues I report to the team leader.
	C10	Colleagues often share work experiences and skills with each other.
Safety management	C11	The company will proactively spend time and money to train me.
	C12	If I sustain a significant injury at work, the company will investigate and handle the matter.
	C13	We can report any violations or misconduct of others by contacting the person in charge at the scene.
	C14	Workers who do a good job in safety will be rewarded.
	C15	The construction site frequently organizes safety training for us to attend.
	C16	When I encounter an emergency situation, I can access emergency supplies to deal with it.
	C17	After a construction accident occurs, the company will promptly carry out emergency rescue measures.
Safety attitude	C18	I understand the meanings of most safety signs.
	C19	For me, safety is not just about slogans. If there are specific instructions or demonstrations on the construction site, I will follow them accordingly.
	C20	During my rest days, I am also willing to participate in safety production-related activities.
	C21	I will take safety education content seriously and study it carefully.
	C22	I believe in the transparency of safety inspections and the handling of accidents announced by the company.
Safety awareness	C23	The team leader’s violation of safety regulations will not attract everyone’s attention.
	C24	It’s okay to break some safety rules in order to save time.
	C25	For me, completing the project on time is more important than paying attention to safety.
	C26	I have more valuable experience than safety regulations.
	C27	I believe that I can make phone calls at any time during work.

## References

[1] Zohar D. Safety climate in industrial organizations: theoretical and applied implications. J Appl Psychol. (1980) 65:96–102. doi: 10.1037/0021-9010.65.1.96, PMID: 7364709

[2] Dedobbeleer N Beland F. A safety climate measure for construction sites. J Saf Res. (1991) 22:97–103. doi: 10.1016/0022-4375(91)90017-p

[3] Barbaranelli C Petitta L Probst TM. Does safety climate predict safety performance in Italy and the USA? Cross-cultural validation of a theoretical model of safety climate. Accid Anal Prev. (2015) 77:35–44. doi: 10.1016/j.aap.2015.01.012 25697669

[4] Chen Y McCab B Hyatt D. A resilience safety climate model predicting construction safety performance. Saf Sci. (2018) 109:434–45. doi: 10.1016/j.ssci.2018.07.003

[5] Pandit B Albert A Patil Y Al-Bayati AJ. Impact of safety climate on hazard recognition and safety risk perception. Saf Sci. (2019) 113:44–53. doi: 10.1016/j.ssci.2018.11.020

[6] Kiani M Asgari M Gohari FA Rezvani Z. Safety climate assessment: a survey in an electric power distribution company. Int J Occup Saf Ergon. (2022) 28:709–15. doi: 10.1080/10803548.2020.1870832 33847238

[7] Gas S. G. Salminen S. (2011). Organizational safety climate: Impact of gender on perception of workplace safety.Int. Perspecth. 61–77.

[8] Gyekye SA Salminen S. Educational status and organizational safety climate: does educational attainment influence workers' perceptions of workplace safety? Saf Sci. (2009) 47:20–8. doi: 10.1016/j.ssci.2007.12.007

[9] Mohammadfam I Ghasemi F Kalatpour O Moghimbeigi A. Constructing a Bayesian network model for improving safety behavior of employees at workplaces. Appl Ergon. (2017) 58:35–47. doi: 10.1016/j.apergo.2016.05.006, PMID: 27633196

[10] Gao R Chan APC Utama WP Zahoor H. Workers' perceptions of safety climate in international construction projects: effects of nationality, religious belief, and employment mode. J Constr Eng Manag. (2017) 143:04016117. doi: 10.1061/(asce)co.1943-7862.0001226

[11] Loosemore M Sunindijo RY Zhang S. Comparative analysis of safety climate in the Chinese, Australian, and Indonesian construction industries. J Constr Eng Manag. (2020) 146:04020129. doi: 10.1061/(asce)co.1943-7862.0001934

[12] Lingard HC Cooke T Blismas N. Properties of group safety climate in construction: the development and evaluation of a typology. Constr Manag Econ. (2010) 28:1099–112. doi: 10.1080/01446193.2010.501807

[13] Choudhry Rafiq M Fang D Lingard H. Measuring safety climate of a construction Company. J Constr Eng Manag. (2009) 135:890–9. doi: 10.1061/(ASCE)CO.1943-7862.0000063

[14] Lestari F Sunindijo RY Loosemore M Kusminanti Y Widanarko B. A safety climate framework for improving health and safety in the Indonesian construction industry. Int J Environ Res Public Health. (2020) 17:7462. doi: 10.3390/ijerph17207462 33066409 PMC7602245

[15] Li QM Ji C Yuan JF Han RR. Developing dimensions and key indicators for the safety climate within China's construction teams: a questionnaire survey on construction sites in Nanjing. Saf Sci. (2017) 93:266–76. doi: 10.1016/j.ssci.2016.11.006

[16] Zhou QA Fang DP Mohamed S. Safety climate improvement: case study in a Chinese construction Company. J Constr Eng Manage. (2011) 137:86–95. doi: 10.1061/(asce)co.1943-7862.0000241

[17] Omidi L Salehi V Zakerian SA Nasl Saraji J. Assessing the influence of safety climate-related factors on safety performance using an integrated entropy-TOPSIS approach. J Ind Prod Eng. (2022) 39:73–82. doi: 10.1080/21681015.2021.1958937

[18] Lim H Kim S Kim Y Son S. Relative importance analysis of safety climate evaluation factors using analytical hierarchical process (AHP). Sustain For. (2021) 13:4212. doi: 10.3390/su13084212

[19] Tang J Zhu HL Liu Z Jia F Zheng XX. Urban sustainability evaluation under the modified TOPSIS based on Grey relational analysis. Int J Environ Res Public Health. (2019) 16:256. doi: 10.3390/ijerph16020256, PMID: 30658429 PMC6352120

[20] Zeng S Fang Z He Y Huang L. An integrated entropy-COPRAS framework for Ningbo-Zhoushan port logistics development from the perspective of dual circulation. Systems. (2022) 10:131. doi: 10.3390/systems10050131

[21] Zhang HJ Lu MK Ke XB Yu SQ Zhao JZ Wu Y. Evaluation model of black-start schemes based on optimal combination weights and improved VIKOR method. Int J Electr Power Energy Syst. (2021) 129:106762. doi: 10.1016/j.ijepes.2021.106762

[22] Jahanshahloo GR Lotfi FH Davoodi AR. Extension of TOPSIS for decision-making problems with interval data: interval efficiency. Math Comput Model. (2009) 49:1137–42. doi: 10.1016/j.mcm.2008.07.009

[23] Zavadskas EK Kaklauskas A Turskis Z Tamosaitiene J. Selection of the effective dwelling house walls by applying attributes values determined at intervals. J Civ Eng Manag. (2008) 14:85–93. doi: 10.3846/1392-3730.2008.14.3

[24] Sayadi MK Heydari M Shahanaghi K. Extension of VIKOR method for decision making problem with interval numbers. Appl Math Model. (2009) 33:2257–62. doi: 10.1016/j.apm.2008.06.002

[25] Zhang L Yang Y Zhao X. SaaS decision-making method based on cloud model. Acta Electron Sin. (2015) 43:987–92.

[26] Ramakrishnan KR Chakraborty S. A cloud TOPSIS model for green supplier selection. Facta Univ Ser Mech Eng. (2020) 18:375–97. doi: 10.22190/fume200307036r

[27] Salabun W Watróbski J Shekhovtsov A. Are MCDA methods Benchmarkable? A comparative study of TOPSIS, VIKOR, COPRAS, and PROMETHEE II methods. Symmetry-Basel. (2020) 12:1549. doi: 10.3390/sym12091549

[28] Wang P Bai XY Wu XQ Yu HJ Hao YY Hu BX. GIS-based random Forest weight for rainfall-induced landslide susceptibility assessment at a humid region in southern China. Water. (2018) 10:1019. doi: 10.3390/w10081019

[29] Gao L Zhou YZ Guo KR Huang Y Zhu XF. Determining the weights of influencing factors of construction lands with a neural network algorithm: a case study based on Ya'an City. Earth Sci Inf. (2021) 14:1973–85. doi: 10.1007/s12145-021-00657-8

[30] Olden JD Jackson DA. Illuminating the "black box": a randomization approach for understanding variable contributions in artificial neural networks. Ecol Model. (2002) 154:135–50. doi: 10.1016/s0304-3800(02)00064-9

[31] Grömping U. Variable importance assessment in regression: linear regression versus random Forest. Am Stat. (2009) 63:308–19. doi: 10.1198/tast.2009.08199

[32] Liu HJ Hu YR. An evaluating method with combined assigning-weight based on maximizing variance. Sci Program. (2015) 2015:1–8. doi: 10.1155/2015/290379

[33] Li D Liu C Gan W. A new cognitive model: cloud model. Int J Intell Syst. (2009) 24:357–75. doi: 10.1002/int.20340

[34] Wang S Li D Shi W Li D Wang X. Cloud model-based spatial data mining. Geogr. Inf. Sci. (2003) 9:60–70. doi: 10.1080/10824000309480589

[35] Öcal ME Oral EL Erdis E Vural G. Industry financial ratios -: application of factor analysis in Turkish construction industry. Build Environ. (2007) 42:385–92. doi: 10.1016/j.buildenv.2005.07.023

[36] Paupério A Severo M Lopes C Moreira P Cooke L Oliveira A. Could the food neophobia scale be adapted to pregnant women? A confirmatory factor analysis in a Portuguese sample. Appetite. (2014) 75:110–6. doi: 10.1016/j.appet.2013.12.023 24406850

[37] Cronbach LJ. Coefficient alpha and the internal structure of tests. Psychometrika. (1951) 16:297–334. doi: 10.1007/BF02310555

[38] Kim NK Rahim NFA Iranmanesh M Foroughi B. The role of the safety climate in the successful implementation of safety management systems. Saf Sci. (2019) 118:48–56. doi: 10.1016/j.ssci.2019.05.008

[39] He CQ McCabe B Jia GS Sun JD. Effects of safety climate and safety behavior on safety outcomes between supervisors and construction workers. J Constr Eng Manag. (2020) 146:04019092. doi: 10.1061/(asce)co.1943-7862.0001735

[40] Chen HH Li HJ Goh YM. A review of construction safety climate: definitions, factors, relationship with safety behavior and research agenda. Saf Sci. (2021) 142:105391. doi: 10.1016/j.ssci.2021.105391

[41] Ning D-c Wang J p Ni G-d. Analysis of Factors Affecting Safety Management in Construction Projects, 2010 International Conference on Management and Service Science, Wuhan, China (2010) 1–5. doi: 10.1109/ICMSS.2010.5576911

[42] Long-chang Z Yan-hong Y Xu-hui Z. SaaS Decision-Making Method Based on Cloud Model[J]. Acta Electronica Sinica, (2015) 43:987–992. doi: 10.3969/j.issn.0372-2112.2015.05.023

